# Hypodermic Medication for Beginners

**Published:** 1881-08

**Authors:** E. A. Cobleigh

**Affiliations:** Athens, Tenn.


ATLANTA
Medical and Surgical Journal.

VOL. XIX.] AUGUST—1881. [No. 5

### Original Communications.

### HYPODERMIC MEDICATION FOR BEGINNERS.

BY E. A. COBLEIGH, M.D., ATHENS, TENN.

I see our professional periodicals every now and then contain articles on the subject of hypodermicism, which is rapidly and deservedly gaining ground among us. And yet there are still hundreds of physicians who have not fallen into line with the progress of the age, and who do not show any indications of so doing. This is particularly the case with hosts of country physicians, some of whom are too indolent to get out of old rules by the adoption of "new fangled" ideas or practices, especially if such action necessitates any study of the subject; while a second-class shrink from it through timidity, engendered by published reports of accidents; and, lastly, some refrain from the frequent use of the hypodermic needle, or abandon it altogether, after once instituting its use, because the syringes get out of order or the solutions spoil.

To the indolent class I have nothing to say, as they are proof against all appeals which disturb them in their regular rotation of pulse-feeling, tongue-inspection and dosing out of drugs in the "good old orthordox way." But for the two latter classes I will make a few comments, and more particularly would I address myself to the class last mentioned, because I have lately read several articles, the object of which were to suggest methods of overcoming the troubles incident to the much used syringe, and the deteriorating solutions.

Three or four years ago I published an essay on this same subject, in one of our journals, and should not venture to repeat it now were it not that in my intercourse with other physicians, in the country, I find many of them holding erroneous views of the matter, satisfying me of the prevalence of much ignorance, elsewhere, as well as in my own immediate neighborhood, touching this subject of hypodermic medication. A goodly number of the older practitioners have never seen one of the injections given, and, therefore, they either do not use them at all, or else they use them bunglingly. To the credit of the more recent graduates (and the faculties of our colleges) I must say that they mostly use the syringe, and use it scientifically and well.

First, then, I will observe that this is one of the conveniences which the rural doctor may reap the benefits of, and use as freely as his sometimes more highly favored city colleague. There is no complicated machinery to necessitate proximity to an instrument maker, no bulk to prevent easy carrying, no delay in getting ready for action, no nice pharmaceutical manipulation required, to demand the services of an experienced apothecary. The whole apparatus, medicines and all, may be carried, as I carry mine, in the vest pocket, and there is *no need* whatever of any spoiling of solutions, to trouble the operator.

Not long since I read the report of a long discussion, before the Baltimore Medical Society, on the subject of how to carry soluble remedies so as to keep, to be strictly accurate in dose, and be ready at all times for administration, and the favorite method proposed by most of the speakers was to have said drugs made up by the apothecary into pellets, these to be extemporaneously dissolved at the moment of using. It was claimed that these were not bulky, would keep well, and *if proper and soluable excipients were used in their manufacture*, would not harden, beyond the point of ready solution, by age. As the correct method of preparing remedies for introduction under the cuticle is the first step in hypodermicism, let us consider this important point right here; and I begin by asserting that to my mind there is no use of carrying the solutions at all, to which there is so many objections for the rural practitioner, and equally no excuse for the manipulations of the apothecary, which are required to get the pellets made according to the formula of our Baltimore brethren. There is too much "red tape" about it, so to speak.

I doubt if I can better make myself understood than by giving my own plan of procedure, and adding such comments as seem indicated. And I preface by saying that nice accuracy, while not always essential, is generally best and certainly safest, in dosing patients hypodermically, and morphine is the drug, typical of all the rest, which I shall use as illustrative of the entire class. The first thing to be done is to procure a syringe, and the finer and more expensive instruments are not at all necessary, though desirable. I have used several makes and styles, my last being a nickel plated one, encasing a glass barrel, with graduated piston, and thumb set-screw. The main points to be looked to are that the needles are fine, sharp, and non-corodable, and that they fit air tight to the syringe. My instrument has two lips, which is advisable, as they are so delicate as to be sometimes unavoidably broken. It rests in a morocco covered case containing a compartment occupied by a vial for holding solutions. And this small space, after removing the bottle, is the magazine wherein I store my hypodermic ammunition, sufficiently roomy to answer every practical purpose.

The instrument once procured requires very little attention, if it be well wrinsed out after every occasion of use, this to be done by repeated filling with and ejection of clean water, by means of the piston. For the country physician there is only one source of trouble and complaint, generally resulting from the non-frequent use of the syringe. By alternations of moisture and dryness the leather packing, on most syringes, sooner or later gets dry, harsh and too small for the barrel, by shrinkage. When this occurs the piston slips loosely, sucks up liquids imperfectly and fails to exclude air from the chambers, so that the administration of injections become worrying to both patient and physician, and may also give rise to unpleasant consequences. But though this very state of affairs has been the cause of throwing aside the instrument altogether in many cases, nothing is simpler than its prevention. This is secured by drawing the piston entirely out of the barrel and depositing it, with the leather downwards, into a graduate, or other suitable vessel, containing a few drops of sweet oil. Let it remain, thus soaking, over night and it is ready for use again next morning by simply wiping off all superfluous oil from the leather washer, and inserting it again into the instrument whence it came. By occasional repetition of this procedure your syringe recuperates while you sleep, and it is ever ready for service without any attention from the instrument maker. One other matter and I leave this branch of my subject. After use the delicate wire which belongs with the points should be carefully re-inserted in the needle so as to penetrate its entire length, otherwise it will sometimes have its calibre obliterated by dirt or rust.

Now, a few words regarding the medicines and their preparation. I go to the drug store and have two grains of morphine weighed out, or weigh it myself on my office scales. This is divided and put into twelve small powder papers, giving me that number of doses of the drug in sixths of a grain. Then two more grains are made into eight powders, giving me eight quarter-grain doses. I have atropine put up in the same way, in 1-100 and 1-50. Other drugs can be so prepared if wished, but many of them need no such nicety of accurate division. F. e. ergot is ready for use in most saddle-bags and medicine chests. Quinine can be guessed off into doses with sufficient precision. Aqua ammonia is already in solution in cases of emergency. It is useless for me to go over the entire list. I carry my powders in the vacant space of my syringe case, which hold many of these little fellows when nicely folded.

Reaching the bedside I am ready to give chloroform, or any other liquid desired, hypodermically, by means of my graduated syringe, in doses to suit me and on the instant. But suppose the remedy needed is atropine, morphia, or other powder. I call for a tumbler of tepid water, and if not to be had I frequently use it cold. On presentation of the water I fill my syringe by suction, throw the rest out of the tumbler, empty one of my ready weighed powders into said glass, and having pushed out a few drops of the water in my instrument to allow of expansion when the powder is dissolved in the remainder, I forcibly eject what is left on to the drug. By working this forcibly in and out of the syringe barrel a few times perfect solution of most of the soluble substance is obtained. The medicine is then sucked up and the needle is then screwed on tight. Now the instrument must be held with the needle up, and the piston gently pressed up until liquid flows by drops from the end of the needle, all air is thus expelled and I am ready for operation.

Right here is a good place for a word regarding distilled water as a menstrum. I never use it. It is a useless refinement. Of course I endeavored to obtain *clear* water in all cases, but not infrequently has the necessity of circumstances forced me to be content with a liquid which would have been said to hazard an abscess. Yet, although I have several times run this risk knowingly, in emergencies, I have never had an abscess to occur in my entire practice, except where the *medicine itself* which I chose to introduce was of such a nature as to render a subsequent abscess unavoidable. Experience has thus taught me that the subdermal cellular tissue is wonderfully tolerant, not only of the presence of irritating substances foreign to it, but also of awkward and rough treatment during manipulation. And these two circumstances should afford comfort and courage to the timid practitioners, who are fearful of inflammations and purulent collections as a result of their treatment, if the use of the syringe be adopted by them.

Well, my instrument is charged, and I am ready for action. My next business is the selection of a site for the puncture, this being usually in the neighborhood of the delloid, unless some special purpose calls for another location. And for general purposes I do not stand on any ceremony regarding the "where" of my injection. All that need enter into consideration here is the selection of a spot rich in cellular tissue and poor in large vessels, nerves and tendinous or fibrous structure. With the left thumb and finger I pinch up a small fold of the loose skin and submit it to considerable pressure between them for an instant. This benumbs the terminal nerve filaments in timid and impressible subjects, obtunding all, or nearly all, pain from the prick of the needle point, and is wholly unnecessary in those cases where sensibility to suffering is not exalted, or, generally speaking, where no fear of the operation exists, either naturally or from previous experience having dispelled it.

This done, I slip my fingers up the surface of the arm a few lines, still, however, grasping the same fold of integument in nearly the same place; with my right hand I bring my instrument to this spot and hold it in a position horizontally with the arm to be injected and parallel thereto, its under side resting lightly upon the skin and below the point of intended insertion. Now, drawing the raised integument forcibly away from the syringe, so as to put it well upon the stretch, I *quickly* push the needle, previously wet, right into the spot where I have pinched the sensation out, and run it easily in (still, parallel with the surface) clear up to the shoulder of the instrument. This is the only part of the performance where nicety is required. Having seen the needle pushed home without either raising the skin, or making it tense, in some cases in an oblique or perpendicular direction, and otherwise with much carelessness, I consider it important to impress upon the beginner the correct and least annoying manner of procedure.

After the needle point has made its entrance, the rest of the operation is practically painless, though a feeling of burning or stinging may follow the injection of the liquid, especially if it be of an acrid character. Nor, indeed, is the pain at all severe in any case, even when the needle enters, except in persons of great susceptibility, who are frightened at a pin scratch or other trifle, and then 'tis but momentary at the most, unless a pretty good sized nerve filament be accidentally injured, and this is rare.

The next step is to partially withdraw the needle, so as to free its orifice from any chance obstruction, occasioned by its penetration of the tissues, and gives a good outlet to its fluid contents This accomplished, the piston is slowly pushed home into the barrel and the instrument withdrawn; a finger being now pressed on the puncture, and the tumefied spot, resulting from the liquid beneath the skin, is gently rubbed by the fingers of the other hand until absorption or disappearance of the elevation occurs. Our operation is now complete; it has taken only a few moments to go through the whole procedure, and the patient is, or very soon will be, getting an effect from our remedy, proportionate to the dose. An irritable stomach cannot interfere to thwart our object; unconsciousness has not debarred us from medication through inability of deglutition; a fastidious or exalted sense of taste cannot reject the nauseous dose; childish obstinacy becomes inert; and lastly, we are not forced to wait an hour or so for results upon which life or death, mayhap in a few minutes, inevitably depend.

A word regarding my ready-weighed powders and I will desist. If I wish to give a dose of 1-16 grain of morphia, it is prepared. Should the desired quantity be less, I dissolve $\frac{1}{4}$ grain in a syringeful of water, set my thumb-screw at 15 minims on the rod, and inject as before, but only force out half the contents of my instrument by this means, eqivalent to $\frac{1}{8}$ grain. For a dose of 1-12, in a child, the same method of using a powder of $\frac{1}{6}$ suggests itself. Where $\frac{1}{4}$ grain is wanted, 'tis ready weighed, double the dose by dissolving two powders at once in same amount of liquid as used for one. So, also, $\frac{1}{3}$ gr. is obtained by means of two powders of the $\frac{1}{6}$, and *ad libitum* to suit the case in hand. Each kind of powder is put up in a colored wrapper different from those used for the other varieties or doses, and each is marked in pencil $\frac{1}{4}$, $\frac{1}{6}$, or otherwise, as the case may be, and "M" for morphia, "A" for atropia, etc., to distinguish them and avoid mistakes.

To professionals of experience who find my essay dry, I would say it is not for experts I write. If they find no new suggestions in it, let them bear in mind my object is not to instruct *them*, but to outline for beginners and especially my rural brethren, and there are hundreds who need such instruction, as my personal experience and observation amply demonstrate—the simplicity, desirability and proper mode of using the hypodermic method of treatment. Once tried according to the foregoing plan, I doubt if a simpler or more satisfactory can be found, or if hypodermicism will thereafter be abandoned.

